# ADHD and political participation: An observational study

**DOI:** 10.1371/journal.pone.0280445

**Published:** 2023-02-21

**Authors:** Israel Waismel-Manor, Yael R. Kaplan, Shaul R. Shenhav, Yair Zlotnik, Shira Dvir Gvirsman, Gal Ifergane

**Affiliations:** 1 Department of Government and Political Theory Division, School of Political Science, University of Haifa, Haifa, Israel; 2 Department of Political Science, The Hebrew University of Jerusalem, Jerusalem, Israel; 3 Department of Neurology, Soroka University Medical Center, Faculty of Health Sciences, Ben Gurion University of the Negev, Beersheba, Israel; 4 Department of Communication, Tel Aviv University, Tel Aviv, Israel; University of Edinburgh, UNITED KINGDOM

## Abstract

**Background and objective:**

Over the past decade, researchers have been seeking to understand the consequences of adult attention-deficit/hyperactivity disorder (ADHD) for different types of everyday behaviors. In this study, we investigated the associations between ADHD and political participation and attitudes, as ADHD may impede their active participation in the polity.

**Methods:**

This observational study used data from an online panel studying the adult Jewish population in Israel, collected prior the national elections of April 2019 (N = 1369). ADHD symptoms were assessed using the 6-item Adult ADHD Self-Report (ASRS-6). Political participation (traditional and digital), news consumption habits, and attitudinal measures were assessed using structured questionnaires. Multivariate linear regression analyses were conducted to analyze the association between ADHD symptoms (ASRS score <17) and reported political participation and attitudes.

**Results:**

200 respondents (14.6%) screened positive for ADHD based on the ASRS-6. Our findings show that individuals with ADHD are more likely to participate in politics than individuals without ADHD symptoms (B = 0.303, SE = 0.10, p = .003). However, participants with ADHD are more likely to be passive consumers of news, waiting for current political news to reach them instead of actively searching for it (B = 0.172, SE = 0.60, p = .004). They are also more prone to support the idea of silencing other opinions (B = 0.226, SE = 0.10, p = .029). The findings hold when controlling for age, sex, level of education, income, political orientation, religiosity, and stimulant therapy for ADHD symptoms.

**Conclusions:**

Overall, we find evidence that individuals with ADHD display a unique pattern of political activity, including greater participation and less tolerance of others’ views, but not necessarily showing greater active interest in politics. Our findings add to a growing body of literature that examines the impact of ADHD on different types of everyday behaviors.

## Introduction

Attention-deficit/hyperactivity disorder (ADHD) is a neuropsychiatric syndrome characterized by marked lack of attention and/or hyperactivity and impulsiveness [1]. These symptoms affect individuals’ ability to function in social and professional contexts. While initially ADHD was considered a childhood condition, an increasing body of literature suggests that it should be recognized as persisting into adolescence and adulthood [2–4]; Indeed, the DSM-5 classifies ADHD as a lifelong condition [5]. Substantial efforts have been devoted to identifying the condition’s prevalence in the adult population (estimates range from 1% to 7.3%) [6–8] and its comorbidities (depression, anxiety, addictions, memory problems, and others) [9–13]. In addition, researchers are working to understand the effects of ADHD on social behavior and achievement. This line of inquiry has found ADHD to be correlated with educational underachievement, unemployment, involvement in motor vehicle accidents, and criminal behavior [14–17].

Political participation—the voluntary actions engaged in by members of the public in the political arena—is considered a cornerstone of functioning democracies, enabling nonprofessionals-citizens to influence public policy and the elected officials who shape those policies [18]. Traditionally, some specific activities have become known as conventional or institutionalized modes of participation [19]. These include actions such as voting, contacting politicians, or participating in a demonstration or a political convention. It is well-established that various factors affect levels of political participation, including age, income, education, and religiosity [20]. Previous work also examined the association between neuropsychiatric conditions and political behavior. For example, Sund et al. (2017) find that various chronic conditions, including epilepsy, dementia, psychotic mental disease, and other degenerative brain diseases, are associated with voting turnout [21]. Others have explored the negative effect of depression on voting [22–25]. Bernardi et al. (2022) indicate a negative relationship between depression and external political efficacy [26]. However, to the best of our knowledge, the association between political participation and ADHD has not previously been studied.

The association between ADHD and political participation deserves more attention both because it is a relatively common condition and because of its distinctive characteristics [27]. Several reasons that raise the expectation that ADHD symptoms will be associated with lower levels of political participation. For example, studies indicate that political participation requires resources such as time, money, and civic skills that comprise communications and organizational capacities [28, 29]. Accordingly, more educated individuals with higher incomes and higher socioeconomic status tend to have more extensive resources, which increase political participation [30, 31]. Simultaneously, studies show that individuals with ADHD are likely to have fewer resources of this kind, as it has been associated with lower educational levels, higher unemployment rates, and lower income [32]. Moreover, ADHD might reduce psychological resources, leaving little time or energy to follow politics and thus accumulate political knowledge [30]. This, in turn, might lower their likelihood of participating politically.

Another known predictor of political participation that can be relevant for ADHD symptoms is political interest [33–35]. It is possible that some ADHD symptoms, such as inattention or impulsivity, are at odds with the willingness to pay attention to current political issues at the expense of other issues. This can be yet another reason to expect lower levels of political participation by individuals with ADHD. However, other ADHD characteristics, such as impulsivity and hyperactivity, could be associated with more intensive, and less restrained, political participation. This is especially true when it comes to the expression of one’s own opinion.

Attentiveness to various political messages in the competitive environment of the political domain raises questions regarding the views of individuals with ADHD on tolerance to multiple opinions and voices in the political sphere. This matter is particularly important since it might conflict with one of democracy’s fundamental qualities—freedom of speech. Hence, an interesting question we can address in our analysis is whether ADHD is associated with intolerance towards freedom of speech and to what extent it reflects on other democratic norms. Lastly, some evidence indicates that individuals with ADHD might show less trust in governmental institutions. For example, Dvorsky et al. (2022) show that lower COVID-19 vaccination willingness and trust were correlated with ADHD among adolescents [36]. As a governmental office approved these vaccinations, we are interested in learning whether individuals with ADHD are less inclined to trust other political institutions, such as parties and parliaments. As individuals with ADHD have needs that can be addressed through public policies beneficial to their condition, such as subsidizing medications or accommodating educational, social, or psychological needs, we are also interested to learn whether they feel less politically represented.

## Methods

### Data collection

The current study is based on a 5-wave online panel study, with a sample representative of the adult Jewish population in Israel. Data for the larger project were collected between January and April 2019, before and after the national elections held on April 9 of that year. The relevant data for this study were gathered in three waves between January 28 and March 29, 2019. Most of the measures were collected during the first wave. News-gathering habits were reported in the second wave. Two measures—symptoms of ADHD and (curbs on) freedom of speech—were reported in the third wave. In case a measurement was collected over more than one wave, we included in our data the first time the question was presented to the participants (a summary of data collection can be found in S1 Appendix).

Respondents were recruited by iPanel, an online research company that maintains an extensive pool of survey participants in Israel who receive gift cards in exchange for their participation in periodic surveys. Before starting the survey, participants signed an online informed consent form. The Ethics Committee of Tel-Aviv University approved this study (IRB approval no. 0000817–1). To assure that our panel is representative of the general population, participants were recruited from the overall iPanel pool based on demographic quotas that approximate these same demographics among the general Israeli population. These included gender, age, geographical location, education, religiosity, and income. As in most recent political studies [37–39], the active sampling process ensures that once a quota is met, no more invitations are sent to that demographic group. A sample of 1,374 participants was recruited and consented to participate in the study. Of these, 1369 panelists completed the relevant questionnaires for this study.

### Measures

#### Symptoms of attention-deficit/hyperactivity disorder (ADHD)

To assess the presence of adult ADHD symptoms, we employed the widely used Adult Self-Report Scale (ASRS) screening tool [40]. Studies suggest that both the ASRS-6 and ASRS-18 offer excellent concordance with clinical diagnoses [41]. We used the shorter version (ASRS-6). Thus, respondents were asked to report how often they had experienced each of six symptoms of adult ADHD over the past six months (e.g., “Is it difficult for you to finish the small details of a project, after you have completed the challenging parts”; “How often do you have difficulty remembering appointments or commitments”). Each item was scored on a 5-point response scale (1 = never; 5 = very often). A composite ADHD index was created by summing the value of each participant’s response scores for all six items (M = 13.19, SD = 3.87, Cronbach’s α = .739). Following previous work, we predefined a dichotomous scoring rule [42], with a score of 17 as our cutoff point (a value widely accepted by practitioners and researchers [43]). Thus, for our main analysis (see below), scores of 6–17 were classified as non-ADHD and scores > 17 (i.e., 18–30) were classified as reflecting ADHD. Thus, throughout the paper, the term ADHD refers to participants screening positive for ADHD using the ASRS-6. Participants were also asked to report whether they were currently using stimulant drugs for the treatment of ADHD.

#### Political participation

*Traditional political participation*. Following similar studies that examined political participation [44, 45], respondents reported whether they had engaged in seven specific actions (1 = no; 2 = yes) during the last year (e.g., “I tried to persuade people to vote for the party I am voting for, or not to vote for the party I oppose”; “I volunteered at a party or candidate’s election headquarters”; “I participated in a demonstration or a political convention”). A composite index of traditional political participation was created by summing the participant’s responses for all seven items (M = 1.50, SD = 1.45, Cronbach’s α = .614).

*Digital political participation*. Due to the prominent role played by the digital sphere in everyday life, including politics, and considering recent research on patterns of social media use among individuals with ADHD [46, 47], we also examined political participation via social media. Specifically, we asked participants how often during the previous week they had engaged in the following activities, on a scale of 1 (never) to 5 (several times a day):

*Connecting with politicians or parties via social media*. This item was measured based on seven related items, including following, reading, sharing, and responding to content coming from politicians or parties. A composite index for this variable was created by averaging responses to the seven items (M = 2.00, SD = 0.82, Cronbach’s α = .846).

*Expressing political opinions on social media*. We asked participants how often they expressed their political views on social media, either by posting their own view or by sharing content posted by a friend. A composite index of the political expressiveness variable was created by averaging responses to the two items (M = 1.59, SD = 0.88, Cronbach’s α = .820).

*Sharing news on social media*. Participants reported their news-sharing habits on social media based on two items: how often they posted a news story on social networks, and how often they shared or distributed a news story posted by others. A composite index of the news-sharing variable was created by averaging responses to the two items (M = 1.73, SD = 0.84, Cronbach’s α = .749).

#### News consumption

News consumption was measured in two ways. First, we asked participants to report on a scale of 1 (not at all) to 5 (regularly) how often they access each of the five most popular print and online media outlets in Israel (Yediot Aharonot, Ynet, Israel Hayom, Walla, and Mako). A composite index for the five outlets was created by averaging the responses (M = 2.77, SD = 0.85, Cronbach’s α = .602).

Second, we assessed participants’ current news-gathering habits; or more specifically, the degree to which their information-gathering was passive rather than active. Respondents were asked how much each of four statements described them on a scale from 1 (not at all) to 5 (very much), where each statement reflected passive news consumption (e.g., “I trust that my friends will let me know what’s important in the news”; “I do not keep track of the news because I know the news will reach me”). A composite index of the “news will find me” items was created by averaging the four responses (M = 2.62, SD = 0.78, Cronbach’s α = .632).

#### Political attitudes

*Sense of political representation*. Sense of representation was operationalized by two dimensions of Pitkin’s classic definition [48], with one item for each: the descriptive (“There is a political party in the country which includes representatives who are similar to me in terms of characteristics and background”) and the substantive (“There is a political party and/or politician in the country who represents my views”). Responses to both were given on a scale from 1 (not at all) to 5 (to a great extent).

*(Curbs on) freedom of speech*. Tracing back our expectation for less tolerance to multiple opinions and voices in the political arena, we were interested here not in support for freedom of speech, but in its opposite: intolerance of differing opinions. Moreover, most people tend to abstractly support freedom of speech and therefore show little variance. This variable is more likely to yield an actual measure of respondents’ attitudes to other points of view. Participants were asked to report their agreement with seven statements on a 7-point scale, where 1 = do not agree at all and 7 = strongly agree (e.g., “Sometimes even in a democracy all kinds of opinions have to be silenced”; “There are situations when it is more important to silence certain opinions than to let everyone express themselves”). A composite index for this variable was created by averaging responses to the seven items (M = 3.64, SD = 1.32, Cronbach’s α = .830).

*(Curbs on) democratic norms*. Here again we measured, not support for democracy, but its opposite: support for violating democratic ideals under certain conditions. Participants were asked to report their agreement with two statements on a 5-point scale, where 1 = strongly disagree and 5 = strongly agree: “To address Israel’s special problems, the country needs a strong leader who will not be inhibited by the Knesset [Israel’s parliament] and the possibility of new elections”; “There are times when it seems better to deviate from the rules of the democratic game in order to achieve significant change.” A composite index was created by averaging responses to the two items (M = 3.32, SD = 1.05, Cronbach’s α = .637).

*Political orientation*. Respondents were asked to place themselves on a political orientation scale ranging from 1 to 7, where 1 indicates the extreme right and 7 the extreme left (M = 3.21, SD = 1.61). (As Hebrew is written from right to left, the scale orientation was in accordance with the two anchors).

*Trust in political institutions*. In three items, participants reported their level of trust in the government, parliament, and politicians on a scale from 1 (no trust at all) to 5 (very high level of trust). Responses were averaged to create a composite index (M = 2.41, SD = 0.75, Cronbach’s α = .833).

*Political interest*. In four items, participants were asked to report on a scale of 1 (strongly disagree) to 5 (strongly agree) their level of interest in politics (e.g., “I have a great deal of knowledge on political issues”; “Political matters are important to me personally”). A composite index was created by averaging responses to the four items (M = 3.53, SD = 0.79, Cronbach’s α = .848). The full wording of all measures can be found in the Online Appendix.

*Covariates*. To test whether ADHD symptoms affect political participation and attitudes above and beyond other potential explanations, we controlled for other known factors. These include sex, age, level of education, level of income, religiosity, and stimulant therapy. As political orientation and interest are also known factors of political participation, we controlled for them as well. Lastly, we controlled for participants’ political knowledge. To measure political knowledge participants were presented three questions concerning current affairs. The scale ranged from 0 (answered all questions wrong) to 3 (answered all questions correctly).

### Statistical analyses

We first examined the association between ADHD symptoms and our outcome variables by comparing the dichotomous non-ADHD and ADHD groups (i.e., ASRS score ≤ 17 or < 17, respectively). Differences in proportions between ADHD and non-ADHD individuals were assessed using Pearson’s chi-square analysis. Next, we divided participants into five groups according to their ASRS-6 scores and tested whether a higher ASRS score is associated with specific political behaviors and attitudes. As a third test, we reran all analyses comparing two groups of respondents: those who scored above 17 in the ASRS-6 but were not taking prescribed ADHD medication; and all respondents who were taking such medication, regardless of their ASRS-6 scores. Finally, to test whether having ADHD symptoms is correlated with political behavior and attitudes above and beyond other factors that might influence the latter, we ran a multivariate linear regression analysis controlling for the following variables: age, sex, level of education, level of income, religiosity, political orientation, political interest, political knowledge, and stimulant therapy. All analyses were conducted using SPSS (Statistical Package for Social Science), Version V25 (IBM® SPSS® Statistics V25, Armonk, USA). The level of statistical significance was set at p < .05.

## Results

A sample of 1,374 participants was recruited for this study. Of these, 1369 panelists completed the relevant questionnaires, 44.0% of whom were female. Two hundred (14.6%) subjects screened positive on the ASRS (i.e., had an ASRS-6 score higher than 17) and were coded as having symptoms of ADHD. While this number is higher than identified in the adult population (estimates range from 1% to 7.3%) [6–8], we believe the difference stems from the fact that our findings are based on self-report and are not a result of neurological tests used to detect the ADHD. Means and standard deviations of the ASRS scores are presented in Table 1. Table 1 also displays the main socio-demographic characteristics of the ADHD and non-ADHD groups (age, sex, education, income, religiosity, and political orientation) as numbers and percentages and each socio-demographic category’s share in the general population. Age (*χ*^2^ = 29.41(4), *p* < .001) and income (*χ*^2^ = 20.63(4), *p* < .001) were correlated with ADHD, while sex, education, religiosity, and political orientation were not (*p*s > .119).

**Table 1:** Characteristics of the study population.

Characteristic	Category	Jewish Population(%)	ADHD, N(%)	Non-ADHD, N(%)	Total N (%)	p value
			200 (14.6)	1169 (85.4)		
**Sex**						
	Male	48.5	112 (56.0)	600 (51.4)	712 (52.0)	0.226
Total					1368 (100.0)	
**Age** (years)						
	18–29	20.2	71 (35.7)	236 (20.2)	307 (22.5)	**0.000**
	30–39	19.7	51 (25.6)	290 (24.8)	341 (24.9)	
	40–49	18.1	38 (19.1)	281 (24.1)	319 (23.3)	
	50–59	14.7	27 (13.6)	199 (17.0)	226 (16.5)	
	60+	27.4	12 (6.0)	162 (13.9)	174 (12.7)	
Total					1367 (100.0)	
**Education**						
	High school or lower	41.0	57 (28.8)	257 (22.1)	314 (23.1)	0.119
	Post-secondary education	18.6	41 (20.7)	255 (22.0)	296 (21.8)	
	Academic degree	40.4	100 (50.5)	649 (55.9)	749 (55.1)	
Total					1359 (100.0)	
**Religiosity**						
	Secular	43.2	112 (56.0)	620 (53.1)	732 (53.5)	0.591
	Traditional	35.4	50 (25.0)	296 (25.3)	346 (25.3)	
	Religious	11.2	27 (13.5)	199 (17.0)	226 (16.5)	
	Ultraorthodox	10.1	11 (5.5)	53 (4.5)	64 (4.7)	
Total					1368 (100.0)	
**Income** (Self-reported, compared to average)						
	Much lower		34 (17.0)	135 (11.5)	169 (12.3)	**0.000**
	Lower		54 (27.0)	213 (18.2)	267 (19.5)	
	Similar		61 (30.5)	376 (32.2)	437 (31.9)	
	Higher		39 (19.5)	381 (32.6)	420 (30.7)	
	Much higher		12 (6.0)	64 (5.5)	76 (5.6)	
Total					1369 (100.0)	
**Political**						
**orientation**	1 (Right)		34 (17.0)	245 (21.0)	279 (20.4)	0.614
	2		31 (15.5)	187 (16.0)	218 (15.9)	
	3		36 (18.0)	209 (17.9)	245 (17.9)	
	4		47 (23.5)	287 (24.6)	334 (24.4)	
	5		33 (16.5)	157 (13.4)	190 (13.9)	
	6		10 (5.0)	52 (4.4)	62 (4.5)	
	7 (Left)		9 (4.5)	32 (2.7)	41 (3.0)	
Total						
**ADHD Symptoms**	ASRS-6 score		19.62	12.09 (2.94)		
Means (SD)			(1.90)			

Abbreviations: SD = standard deviation; ADHD = attention-deficit/hyperactivity disorder; ASRS = ADHD Self-Report Scale.

Notes: ADHD symptoms were assessed using the ASRS-6. Answers were summed to give each individual a total score from 6 to 30. Participants with a score above 17 were screened as positive for the possible presence of ADHD. P values were computed by means of chi-square tests. Level of significance was set at p ≤ 0.05. Significant p values are in bold.

**Fig 1:**
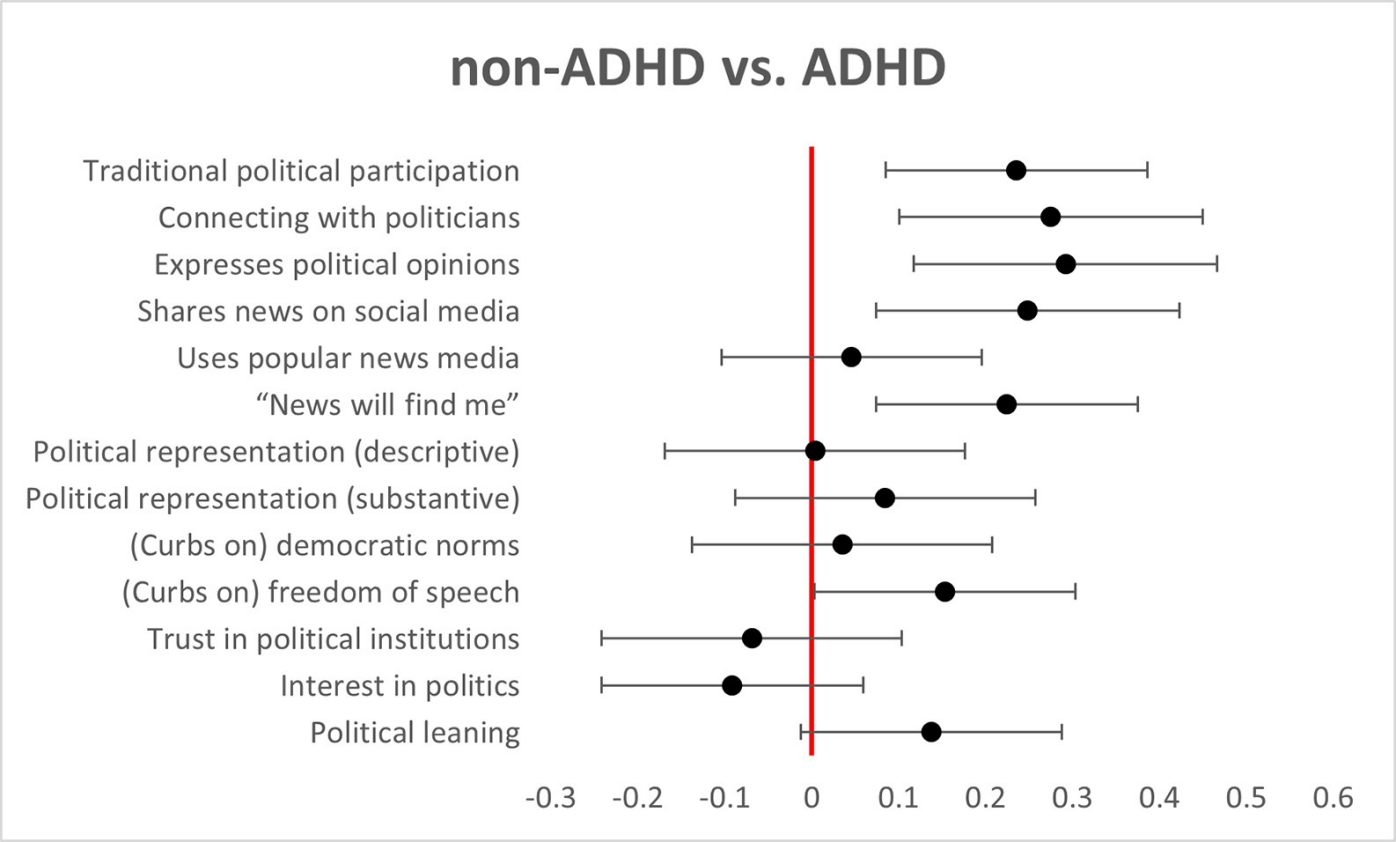
The differences between participants with and without ADHD. Note. Individuals who screened positive for ADHD reported higher levels of political participation than individuals who screened negative, both digitally and in traditional ways. Participants with ADHD had a greater tendency to be passive consumers of news and were also less tolerant towards others voicing their opinions. We did not observe a significant difference between participants with vs. without ADHD in their sense of representation, willingness to curb democratic norms, trust in political institutions, interest in politics, or consumption of popular news media.

Table 2 displays the associations between respondents’ political behaviors and attitudes and the results of the dichotomous ASRS-6 screening. Overall, individuals who screened positive for ADHD reported higher levels of political participation than individuals who screened negative, both digitally (e.g., expressing political opinions on social media: *t*(1013) = 2.781, *p* = .006, Cohen’s *d* = .292) and in traditional ways, *t*(1367) = 3.077, *p* = .002, Cohen’s *d* = .235. At the same time, participants with ADHD had a greater tendency to be passive consumers of news—i.e., waiting for political news to find them rather than actively seeking it out, *t*(1364) = 2.922, *p* = .004, Cohen’s *d* = .224. Respondents with ADHD were also less tolerant towards others voicing their opinions, *t*(1367) = 2.004, *p* = .045, Cohen’s *d* = .153. We did not observe a significant difference between participants with vs. without ADHD in their sense of representation, willingness to curb democratic norms, trust in political institutions, general interest in politics, or consumption of popular news media. Fig 1 summarizes the differences between participants with and without ADHD on these dimensions. As a robustness check, we averaged participants’ responses to all collected measures that appeared in more than one wave (i.e., digital political participation) and reran all the analyses. Results remained directionally consistent and statistically significant (see S2 Appendix).

**Table 2:** Political attitudes and participation patterns of the study population—dichotomous analysis (ADHD vs. Non-ADHD).

Measures, Means (SE)				
Behavior \ Attitude	Scale	ADHD	Non-ADHD	p Value
		N = 200 (13.6)	N = 1169 (86.4)	
		Mean (SE)	Mean (SE)	
**Political participation**				
**Traditional political participation**	Scale: 0–7 (0 = None, 7 = All)	1.79 (1.54)	1.45 (1.43)	**.002**
Total		1369 (100.0), M 1.00 (IRQ 0.00–2.00)		
**Connecting with politicians via social media**	Scale: 1–5 (1 = Never, 5 = Several times a day)	2.19 (.093)	1.96 (0.80)	**.006**
Total		1015 (100.0), M 1.85 (IRQ 1.25–2.55)		
**Expressing political opinions on social media**	Scale: 1–5 (1 = Never, 5 = Several times a day)	1.81 (1.06)	1.55 (0.83)	**.006**
Total		1015 (100.0), M 1.00 (IRQ 1.00–2.00)		
**Sharing news on social media**	Scale: 1–5 (1 = Never, 5 = Several times a day)	1.91 (0.94)	1.70 (0.82)	**.012**
Total		1015 (100.0), M 1.50 (IRQ 1.00–2.00)		
**News consumption**				
**Accessing popular news media**	Scale: 1–5 (1 = Not at all, 5 = Regularly)	2.81 (0.81)	2.77 (0.86)	.549
Total		1369 (100.0), M 2.75 (IRQ 2.25–3.50)		
**Passive news-gathering habits (“news will find me”)**	Scale: 1–5 (1 = Not at all, 5 = regularly)	2.77 (0.77)	2.59 (0.77)	**.004**
Total		1366 (100), M 2.50 (IRQ 2.00–3.25)		
**Political attitudes**				
**Political representation (descriptive)**	Scale: 1–5 (1 = Not at all, 5 = To a great extent)	3.36 (0.99)	3.36 (0.93)	.965
Total		1030 (100), M 3.00 (IRQ 3.00–4.00)		
**Political representation (substantive)**	Scale: 1–5 (1 = Not at all, 5 = To a great extent)	3.44 (0.90)	3.37 (0.90)	.338
Total		1030 (100), M 3.00 (IRQ 3.00–4.00)		
**(Curbs on) democratic norms**	Scale: 1–5 (1 = Strongly disagree, 5 = Strongly agree)	3.35 (1.08)	3.31 (1.04)	.691
Total		1030 (100), M 3.50 (IRQ 2.50–4.00)		
**(Curbs on) freedom of speech**	Scale: 1–7 (1 = Strongly disagree, 7 = Strongly agree)	3.81 (1.28)	3.61 (1.32)	**.045**
Total		1030 (100), M 3.57 (IRQ 2.71–4.57)		
**Trust in political institutions**	Scale: 1–5 (1 = No trust at all, 5 = Very high level of trust)	2.37 (0.69)	2.42 (0.76)	.434
Total		1030 (100), M 2.33 (IRQ 2.00–3.00)		
**Political interest**	Scale: 1–5 (1 = Strongly disagree, 5 = Strongly agree)	3.47 (0.86)	3.54 (0.78)	.234
Total		1367 (100.0), M 3.50 (IRQ 3.00–4.00)		

Abbreviations: M = median; IRQ = interquartile range.

Notes: Table 2 shows the associations between participants’ political behaviors and attitudes and their ASRS-6 questionnaire scores. P values were computed by means of t-tests. Level of significance was set at p ≤ 0.05. Significant p-values are in bold.

We categorized participants dichotomously as screening positive or negative for ADHD based on the widely accepted cutoff score of 17 [43]. However, the appropriate cutoff for diagnosing ADHD among adults remains a topic of debate [5, 49]. More important, in practice ADHD is not necessarily dichotomous, but may vary in its severity. Therefore, to test the robustness of our findings to this more continuous perspective, we also ran additional analyses, in which the ADHD variable was measured on a continuous scale (ranging from 6–30). A linear regression showed results consistent with our primary analysis of the binary measure of ADHD; for example, we found a positive correlation between ADHD and traditional political participation (β = .113, p. = < .001). Results were consistent with other dependent variables discussed above (see S3 Appendix). Furthermore, we divided participants into five similarly sized groups based on their ASRS-6 score: low (6–9), moderately low (10–12), moderate (13–14), high (15–16), and extremely high (17–30). We then retested each measure using ANOVA analyses. While we did not observe a statistically significant difference in participation between each pair of groups, the overall trend was consistent with the binary and continuous measures of ADHD: analysis indicated that people who experience more sever ADHD symptoms report higher levels of political participation (both traditional and digital), a tendency to be passive consumers of news, and intolerance of speech (support for silencing other opinions). To give the reader a sense of the size of the effect of ADHD on various political behaviors, we calculated Cohen’s d. For example, for traditional political participation, the Cohen’s d was .273 for the comparison between “extremely high” and “low.” Such an effect size would be commonly considered as small-medium [50] and is typical for research in social sciences [51]. The results of the ANOVA analyses are presented in Table 3.

**Table 3:** Political attitudes and participation patterns of the study population—continuous analysis (ANOVA).

			N	Means (SE)	95% Confidence interval for mean		p Value
					Lower bound	Upper bound	
	**Political participation**						
**Traditional political participation**							
Low (6–9)			245	1.29 (1.42)	1.11	1.47	.013
Moderately low (10–12)			384	1.42 (1.48)	1.28	1.57	
Moderate (13–14)			266	1.49 (1.40)	1.32	1.66	
High (15–16)			204	1.65 (1.43)	1.45	1.85	
Extremely high (17–30)			270	1.69 (1.49)	1.51	1.85	
Total			1369				
	**Connecting with politicians via social media**						
Low (6–9)			177	1.88 (0.82)	1.76	2.00	.011
Moderately low (10–12)			289	1.93 (0.78)	1.84	2.02	
Moderate (13–14)			190	2.00 (0.80)	1.89	2.12	
High (15–16)			156	2.03 (0.82)	1.90	2.16	
Extremely high (17–30)			203	2.16 (0.90)	2.03	2.28	
Total			1015				
	**Expressing political opinions on social media**						
Low (6–9)			177	1.40 (0.76)	1.29	1.52	< .001
Moderately low (10–12)			289	1.61 (0.86)	1.51	1.71	
Moderate (13–14)			190	1.46 (0.73)	1.35	1.56	
High (15–16)			156	1.68 (0.89)	1.54	1.82	
Extremely high (17–30)			203	1.77 (1.05)	1.63	1.92	
Total			1015				
	**Sharing news on social media**						
Low (6–9)			177	1.63 (0.79)	1.52	1.75	.025
Moderately low (10–12)			289	1.75 (0.83)	1.65	1.85	
Moderate (13–14)			190	1.62 (0.71)	1.51	1.72	
High (15–16)			156	1.82 (0.88)	1.68	1.96	
Extremely high (17–30)			203	1.84 (0.95)	1.71	1.97	
Total			1015				
	**News consumption**						
	**Accessing popular news media**						
Low (6–9)			245	2.82 (0.90)	2.71	2.94	.667
Moderately low (10–12)			384	2.74 (0.85)	2.66	2.83	
Moderate (13–14)			266	2.78 (0.85)	2.67	2.88	
High (15–16)			204	2.72 (0.87)	2.60	2.84	
Extremely high (17–30)			270	2.80 (0.82)	2.70	2.90	
Total			1369				
	**Passive news-gathering (“news will find me”)**						
Low (6–9)			245	2.51 (0.78)	2.41	2.61	.001
Moderately low (10–12)			384	2.54 (0.80)	2.45	2.62	
Moderate (13–14)			266	2.66 (0.75)	2.57	2.75	
High (15–16)			204	2.67 (0.73)	2.57	2.77	
Extremely high (17–30)			270	2.75 (0.76)	2.66	2.84	
Total			1369				
	**Political attitudes**						
	**Political representation (descriptive)**						
Low (6–9)			180	3.33 (0.98)	3.19	3.48	.876
Moderately low (10–12)			293	3.35 (0.91)	3.24	3.45	
Moderate (13–14)			193	3.36 (0.89)	3.24	3.49	
High (15–16)			159	3.43 (0.96)	3.28	3.59	
Extremely high (17–30)			205	3.35 (0.98)	3.21	3.48	
Total			1030				
	**Political representation (substantive)**						
Low (6–9)			180	3.42 (0.88)	3.29	3.55	.295
Moderately low (10–12)			293	3.31 (0.92)	3.20	3.42	
Moderate (13–14)			193	3.41 (0.88)	3.28	3.54	
High (15–16)			159	3.48 (0.86)	3.35	3.62	
Extremely high (17–30)			205	3.33 (0.93)	3.20	3.46	
Total			1030				
	**(Curbs on) democratic norms**						
Low (6–9)			180	3.28 (1.14)	3.11	3.44	.959
Moderately low (10–12)			293	3.33 (1.03)	3.21	3.45	
Moderate (13–14)			193	3.35 (1.00)	3.21	3.49	
High (15–16)			159	3.33 (1.05)	3.17	3.50	
Extremely high (17–30)			205	3.30 (1.05)	3.15	3.44	
Total			1030				
	**(Curbs on) freedom of speech**						
Low (6–9)			245	3.43 (1.46)	3.25	3.62	.036
Moderately low (10–12)			384	3.62 (1.33)	3.49	3.75	
Moderate (13–14)			266	3.66 (1.27)	3.50	3.81	
High (15–16)			204	3.80 (1.25)	3.52	3.87	
Extremely high (17–30)			270	3.80 (1.26)	3.64	3.95	
Total			1369				
	**Trust in political institutions**						
Low (6–9)			180	2.46 (0.93)	2.32	2.59	.340
Moderately low (10–12)			293	2.39 (0.69)	2.31	2.47	
Moderate (13–14)			193	2.43 (0.72)	2.33	2.54	
High (15–16)			159	2.48 (0.72)	2.36	2.59	
Extremely high (17–30)			205	2.33 (0.70)	2.23	2.42	
Total			1030				
	**Political interest**						
Low (6–9)			245	3.56 (0.84)	3.45	3.66	.239
Moderately low (10–12)			384	3.58 (0.79)	3.50	3.66	
Moderate (13–14)			266	3.53 (0.71)	3.45	3.62	
High (15–16)			204	3.50 (0.73)	3.39	3.60	
Extremely high (17–30)			268	3.44 (0.86)	3.34	3.55	
Total			1367				

Note. Participants were divided into five similarly sized groups based on their ASRS-6 scores: low (6–9), moderately low (10–12), moderate (13–14), high (15–16), and extremely high (17–30). P values were computed by means of one-way ANOVA tests. Level of significance was set at p ≤ 0.05. CI = 95% confidence interval.

**Fig 2:**
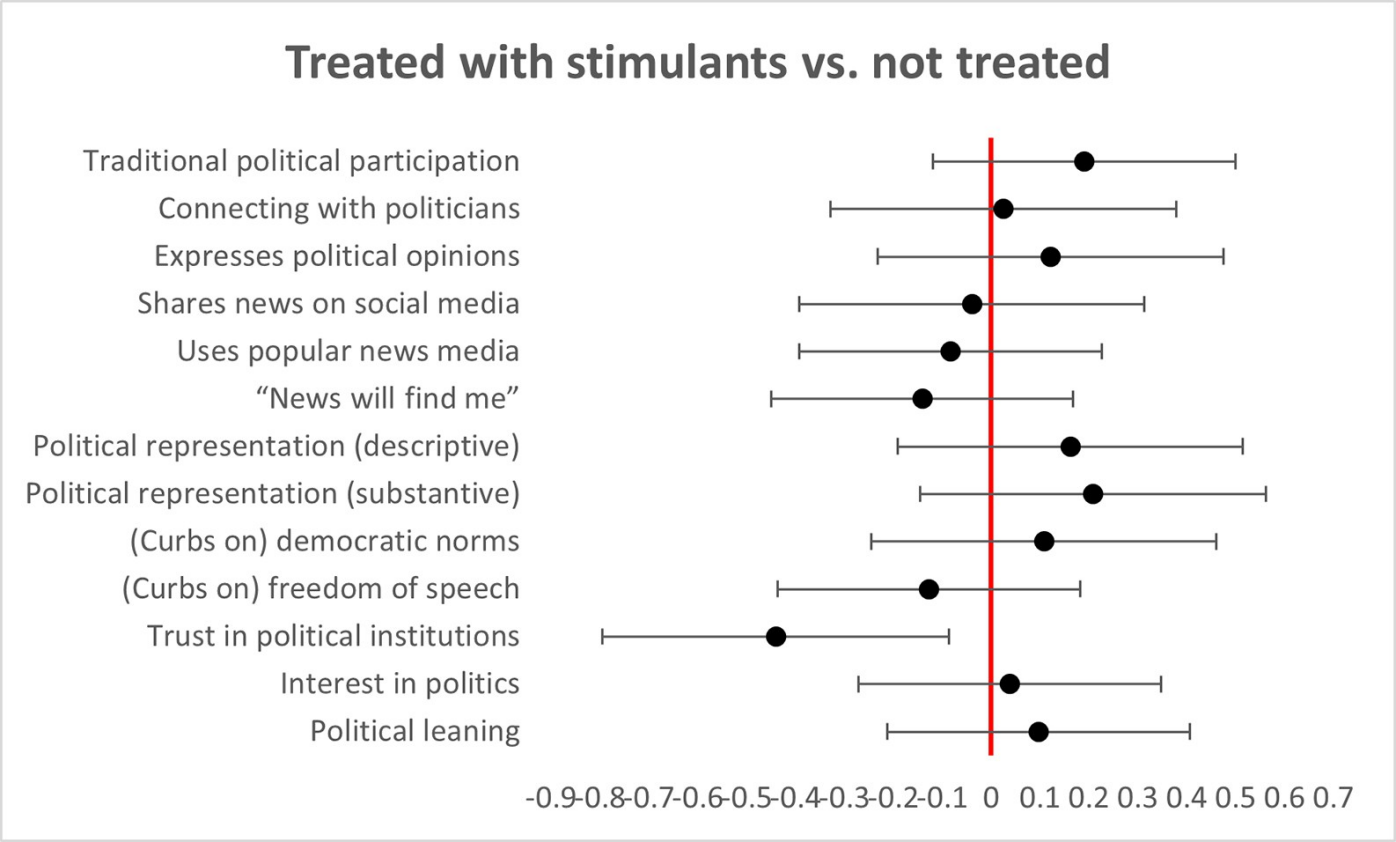
Note. Individuals who screened positive for ADHD but were not taking stimulant drugs to treat their symptoms showed no significant differences in their political behaviors and attitudes compared to all respondents who reported taking stimulants regardless of their ASRS-6 scores in any parameter except trust in political institutions.

Our third set of analyses compared respondents who screened positive for ADHD in our study but were not taking stimulant drugs to treat their symptoms (N = 179), and all respondents who reported taking stimulants regardless of their ASRS-6 scores (N = 52). Several pharmacological agents are used for the treatment of ADHD. Those therapies attempt to reduce the symptoms of patients and to improve their function. We attempted to evaluate whether the use of such medications is associated with different political participation patterns in subjects who present ADHD symptoms. The results showed no significant differences between the two groups in any parameter except trust in political institutions (see Fig 2).

Finally, we ran a set of multiple regression analyses to test the relationship between ADHD symptoms and political behaviors and attitudes while controlling for various factors, including age, sex, education, income, religiosity, political orientation, and use of stimulant drugs. Education, income, religiosity, and age group variables were dummy coded before being added to the regression.

Multivariate regressions confirmed the associations found in the t-tests regarding political behaviors and attitudes (see Table 4). Specifically, ADHD symptoms emerged as a positive predictor of political participation, including traditional participation (B = 0.264, SE = 0.14, p = .021) and the three measures of digital political participation: connecting with politicians via social media (B = 0.181, SE = 0.76, p = .017), expressing political opinions on social media (B = 0.276, SE = 0.80, p = .001), and sharing news on social media (B = 0.254, SE = 0.77, p = .001). ADHD symptoms also predicted passive news consumption (B = 0.163, SE = 0.61, p = .008) and intolerance toward other opinions (B = 0.218 SE = 0.58, p = .034) above and beyond the control variables. No difference between the ADHD and non-ADHD groups was found in regard to the other political actions and attitudes.

**Table 4:** Multilevel regressions.

	B	Std. Error	β	*p value*
**Political participation**				
Traditional political participation	.303	.102	.073	**.003**
Connecting with politicians via social media	.193	.069	.082	**.005**
Expressing political opinions on social media	.289	.076	.116	**< .001**
Sharing news on social media	.266	.076	.111	**< .001**
**News consumption**				
Accessing popular news media	.094	.066	.038	.155
Passive news-gathering (“news will find me”)	.172	.060	.077	**.004**
**Political attitudes**				
Political representation (descriptive)	.046	.083	.017	.538
Political representation (substantive)	.109	.078	.042	.165
(Curbs on) democratic norms	-.041	.087	-.014	.637
(Curbs on) freedom of speech	.226	.103	.060	**.029**
Trust in political institutions	.058	.065	.027	.375
Political interest	-.043	.054	-.019	.435

Note. All analyses control for sex, age, education, income, religiosity, political orientation, and stimulant therapy. Associations between political behavior and attitudes and positive ASRS questionnaire evaluated by linear regression models. P values were computed by multivariate regression. Education, religiosity, income, and age group variables were dummy coded before being added to the regression. Reference categories for the education, religiosity, income, and age variables were an academic level of education, secular, average income, and 30–39, respectively. The level of significance was set at p ≤ 0.05. Significant p-values are in bold.

## Discussion

Previous work suggests that genetic and biological factors might help explain some political behaviors [52–55, 59]. This study examines whether and how one of the most prevalent neuropsychiatric disorders, ADHD [56–58], is correlated with measures of political participation and attitudes. We screened adult participants in a political participation study for ADHD symptomatology using the ASRS-6 screening questionnaire and compared political participation patterns and attitudes of participants who screened positive for ADHD to those of participants who screened negative. In our sample, where the prevalence of ADHD based on the ASRS-6 was 14.6%, we found that positive ADHD screening was associated with higher political participation through both physical and digital channels. However, while ADHD-positive participants tended to express their political opinions via social media, they did not report greater interest in politics or higher levels of active news consumption. Instead, the analysis demonstrated that individuals with ADHD symptoms are more likely to take a “political news will find me” approach. In this sense, our results align with previous work that finds that individuals who suffer from other health conditions in their daily lives tend to participate more regularly in political activity, such as contacting a politician or signing a petition [31].

Additionally, participants with ADHD symptoms were found to be less tolerant of other people’s views. Considering that participants with ADHD symptoms were not more likely to curb democratic norms as a whole, this might reflect their attentiveness rather than a broader democratic issue.

To the best of our knowledge, the impact of ADHD on political behavior has not previously been evaluated. However, a recent study has addressed the use of social media among patients with ADHD. Social media users with ADHD were found to be less agreeable, to post more often, and to use more negations, hedging, and swear words. ADHD is also correlated with addictive social media use [47]. Social media activity in general is rewarding for ADHD patients, as it provides immediate feedback and offers an easy distraction from other tasks. In this sense, political participation through social media platforms is equally rewarding for patients with ADHD.

Impatience and intolerance towards the opinion of others and/or willingness to interrupt others while speaking are also symptomatic of ADHD as defined by the DSM-V [5]. Our findings indicate that this trait is also applicable to the political arena, with participants who screened positive for ADHD displaying lower tolerance towards opposing opinions.

While there was no difference between the ADHD and non-ADHD groups in regard to the amount of political content they consume via popular news outlets, we found that participants with ADHD are more prone to consume news passively, waiting for it to “find them.” This implies, in turn, that these individuals tend to base their current political knowledge on information that is screened for them by others, or that is filtered and curated by social media algorithms. This finding, which was not previously reported, may have implications for how patients with ADHD perceive reality and their vulnerability to being captured by information bubbles.

Participants who were treated by stimulants did not differ from non-treated ADHD-positive participants in our study. A possible explanation for this finding is that pharmacological treatments for ADHD affect symptoms over a limited timeframe, even when long-acting agents are used. ADHD patients tend to take their medication in the morning, so as to manage their symptoms during working hours. However, they are more likely to post on social media at night [47]. As such, their political activity may take place largely at times of day when they are not medically treated.

Our study has four main limitations: First, it was performed on a small population in one specific political context (the state of Israel, which is considered highly polarized). It is, therefore, difficult to draw general conclusions regarding other countries. At the same time, attention disorders are common worldwide, and we hope that further research will this matter in other countries. Second, this study used a screening tool rather than a clinical diagnosis. Third, it is possible that individuals with ADHD will demonstrate a different pattern of responding to surveys. For example, they might lose interest in the middle of filing the survey, depending on the time of day. As no previous literature on this matter exists, our research was carried out using the conventional method without special adjustment for attention disorders. Forth, it examined political participation through self-reports.

Nonetheless, our findings provide insights into the possible effects of ADHD on political behavior. With growing recognition of the existence and impact of ADHD among adults, the effects of the disorder on all aspects of human life are beginning to unfold. Considering that political participation entails voluntary actions taken by individuals to influence public policy and those elected officials who shape those policies, and given that ADHD is correlated with weakened populations, it is important to understand both whether the voices of those with ADHD are heard, and how this segment of society affects the polity.

More broadly, as our understanding and acceptance of neurodiversity grows [59], we need to pay more attention to how various common neurodevelopmental disorders shape our society. The political arena in democratic societies is formed and shaped by all citizens, including “neuro-minorities,” and academic research should address their participation as part of an effort both to improve the social functioning of neurodivergent individuals and to enhance the political system for the benefit of all. Future research is needed to further validate and strengthen our findings, possibly using validated clinical diagnoses and evaluating digital political behaviors via actual inspection of participants’ social media accounts using automated approaches.

## Supporting information

S1 Appendix Measurements collection over waves. (DOCX)

S2 Appendix Analyses results. (DOCX)

S3 Appendix Continuous analyses results. (DOCX)

S1 File (SPS)

S2 File (ZSAV)
