# Supplementary material for: ADHD and political participation: An observational study
Source: PLoS One. 2023 Feb 21;18(2):e0280445. doi: 10.1371/journal.pone.0280445 (PMC9942958; doi:10.1371/journal.pone.0280445)
Supplement: S1 Appendix — (DOCX) [file pone.0280445.s001.docx]

S1 Appendix A: measurements collection over waves.

|  | Scale | Wave 1  28.01.2019-17.02.2019 | Wave 2  27.02.2019-  06.03.2019 | Wave 3  25.03.2019-29.03.2019 |
| --- | --- | --- | --- | --- |
| ASRS-6 | Scale: 1–5 (1=Never, 5=Very often) |  |  | ✔️ |
| Traditional Political Participation | Scale: 1 or 2  (1=I have not, 2=I have) | ✔️ |  |  |
| Connecting with politicians | Scale: 1–5 (1=Never, 5=Several times a day) | ✔️ | ✔️ | ✔️ |
| Expresses political opinions | Scale: 1–5 (1=Never, 5=Several times a day) | ✔️ | ✔️ |  |
| Shares news on social media | Scale: 1–5 (1=Never, 5=Several times a day) | ✔️ | ✔️ |  |
| Uses popular news media | Scale: 1-5  (1=not at all, 5=regularly) | ✔️ |  |  |
| “News will find me” | Scale: 1-5 (1=not at all, 5=very much) |  | ✔️ |  |
| Political representation (descriptive) | Scale: 1–5 (1=Not at all, 5=To a great extent) | ✔️ | ✔️ |  |
| Political representation (substantive) | Scale: 1–5 (1=Not at all, 5=To a great extent) | ✔️ | ✔️ |  |
| (Curbs on) democratic norms | Scale: 1-5 (1=Totally disagree, 5= Strongly agree) | ✔️ |  |  |
| (Curbs on) freedom of speech | Scale: 1-7 (1=Totally disagree, 7 = Strongly agree) |  |  | ✔️ |
| Trust in political institutions | Scale: 1-5  (1=no trust at all, 5=very high level of trust) | ✔️ |  |  |
| Interest in politics | Scale: 1-5 (1=Totally disagree, 5= Strongly agree) | ✔️ |  |  |
| Political leaning | Scale: 1-7 1=Right, 7= (Left) | ✔️ |  |  |
| Political Knowledge | Scale: 0-3 (0=answered all questions wrong, 3= answered all questions correctly) | ✔️ | ✔️ | ✔️ |
