# Supplementary material for: ADHD and political participation: An observational study
Source: PLoS One. 2023 Feb 21;18(2):e0280445. doi: 10.1371/journal.pone.0280445 (PMC9942958; doi:10.1371/journal.pone.0280445)
Supplement: S2 Appendix — (DOCX) [file pone.0280445.s002.docx]

S2 Appendix B: analyses results for all measures that were collected over more than one wave

| **Measures, Means (SE)** | | | |  |
| --- | --- | --- | --- | --- |
| **Behavior \ Attitude** | **Scale** | **ADHD**  **N=200 (13.6)**  **Mean (SE)** | **Non-ADHD**  **N=1169 (86.4)**  **Mean (SE)** | **p Value** |
| **Political participation** |  |  |  |  |
| **Connecting with politicians via social media – W1** | Scale: 1–5 (1=Never, 5=Several times a day) | 2.19 (.093) | 1.96 (0.80) | **.006** |
| Total |  | 1015 (100.0), M 1.85 (IRQ 1.25-2.55) | |  |
| **Connecting with politicians via social media – W1+W2+W3** | Scale: 1–5 (1=Never, 5=Several times a day) | 2.25 (.083) | 2.04 (0.73) | **.001** |
| Total |  | 1354 (100.0), M 1.98 (IRQ 1.50-2.56) | |  |
| **Expressing political opinions on social media – W1** | Scale: 1–5 (1=Never, 5=Several times a day) | 1.81 (1.06) | 1.55 (0.83) | **.006** |
| Total |  | 1015 (100.0), M 1.00 (IRQ 1.00-2.00) | |  |
| **Expressing political opinions on social media – W1+W2** | Scale: 1–5 (1=Never, 5=Several times a day) | 1.77 (1.02) | 1.56 (0.80) | **.007** |
| Total |  | 1342 (100.0), M 1.25 (IRQ 1.00-2.00) | |  |
| **Sharing news on social media – W1** | Scale: 1–5 (1=Never, 5=Several times a day) | 1.91 (0.94) | 1.70 (0.82) | **.012** |
| Total |  | 1015 (100.0), M 1.50 (IRQ 1.00-2.00) | |  |
| **Sharing news on social media – W1+W2** | Scale: 1–5 (1=Never, 5=Several times a day) | 1.79 (0.91) | 1.61 (0.77) | **.014** |
| Total |  | 1342 (100.0), M 1.50 (IRQ 1.00-2.00) | |  |
| **Political attitudes** |  |  |  |  |
| **Political representation (descriptive) – W1** | Scale: 1–5 (1=Not at all, 5=To a great extent) | 3.36 (0.99) | 3.36 (0.93) | .965 |
| Total |  | 1030 (100), M 3.00 (IRQ 3.00-4.00) | |  |
| **Political representation (descriptive) – W1+W2** | Scale: 1–5 (1=Not at all, 5=To a great extent) | 3.38 (0.83) | 3.43 (0.81) | .370 |
| Total |  | 1368 (100), M 3.50 (IRQ 3.00-4.00) | |  |
| **Political representation (substantive) – W1** | Scale: 1–5 (1=Not at all, 5=To a great extent) | 3.44 (0.90) | 3.37 (0.90) | .338 |
| Total |  | 1368 (100), M 3.00 (IRQ 3.00-4.00) | |  |
| **Political representation (substantive) – W1+W2** | Scale: 1–5 (1=Not at all, 5=To a great extent) | 3.51 (0.82) | 3.47 (0.83) | .548 |
| Total |  | 1368 (100), M 3.50 (IRQ 3.00-4.00) | |  |
|  |  |  | |  |

Abbreviations: M = median; IRQ = interquartile range.

Notes: Table X shows the associations between participants’ political behaviors and attitudes and their ASRS-6 questionnaire scores for both the first wave the question was presented and for the averaged responses given over all relevant waves. As can be seen, results remained directionally consistent and statistically significant. P values were computed by means of t-tests. Level of significance was set at p ≤ 0.05. Significant p-values are in bold.
