# Supplementary material for: ADHD and political participation: An observational study
Source: PLoS One. 2023 Feb 21;18(2):e0280445. doi: 10.1371/journal.pone.0280445 (PMC9942958; doi:10.1371/journal.pone.0280445)
Supplement: S3 Appendix — (DOCX) [file pone.0280445.s003.docx]

S3 Appendix C: Continuous Analyses Results

Linear regression analyses indicate a positive correlation between traditional political participation and ADHD symptoms (β = .113, p. < .001); a positive correlation between digital political participation and ADHD symptoms (contacting politicians over social media: *β* = .118, *p*. < .001; expressing political opinions on social media: *β* = .122, *p*. < .001; sharing news on social media: *β* = .086, *p*. = .006). Participants with ADHD had a greater tendency to be passive consumers of news—i.e., waiting for political news to find them rather than actively seeking it out (*β* = .117, *p*. = < .001). Respondents with ADHD were also less tolerant towards others voicing their opinions (*β* = .094, *p*. = .001). We did not observe a significant correlation between ADHD symptoms and participants’ sense of representation, willingness to curb democratic norms, trust in political institutions, or consumption of popular news media (*p*s > .191).
